# The ribosomal exit tunnel as a target for optimizing protein expression in *Escherichia coli*

**DOI:** 10.1002/biot.201100198

**Published:** 2011-11-11

**Authors:** Lydia M Contreras-Martinez, Jason T Boock, Jan S Kostecki, Matthew P DeLisa

**Affiliations:** 1 School of Chemical and Biomolecular Engineering, Cornell UniversityIthaca, NY, USA; 2 Department of Biomedical Engineering, Cornell UniversityIthaca, NY, USA

**Keywords:** Bacterial protein expression, Co-translational protein folding, Molecular chaperones, Translational quality control

## Abstract

The folding of many cellular proteins occurs co-translationally immediately outside the ribosome exit tunnel, where ribosomal proteins and other associated factors coordinate the synthesis and folding of newly translated polypeptides. Here, we show that the large subunit protein L29, which forms part of the exit tunnel in *Escherichia coli*, is required for the productive synthesis of an array of structurally diverse recombinant proteins including the green fluorescent protein (GFP) and an intracellular single-chain Fv antibody. Surprisingly, the corresponding mRNA transcript level of these proteins was markedly less abundant in cells lacking L29, suggesting an unexpected regulatory mechanism that links defects in the exit tunnel to the expression of genetic information. To further highlight the importance of L29 in maintaining protein expression, we used mutagenesis and selection to obtain L29 variants that enhanced GFP expression. Overall, our results suggest that the ribosomal exit tunnel proteins may be key targets for optimizing the overproduction of active, structurally complex recombinant proteins in bacterial cells.

See accompanying Commentary by Baneyx DOI: 10.1002/biot.201100488

## 1 Introduction

The ribosomal exit tunnel was first discovered in eubacteria nearly two decades ago [1]. Since that time, biochemical and genetic analyses have revealed that the tunnel is 100 Å long, extending from the peptidyl transferase center (PTC) to the solvent side of the large ribosomal subunit, and ranges in diameter from ∼10 to 28 Å along its length. It is comprised primarily of 23S rRNA along with four ribosomal proteins (r-proteins), namely L4, L22, L23, and L29. Of these, L4 and L22 are the best characterized owing to their involvement in resistance to macrolides such as erythromycin [2]. Recently, however, much attention has been given to r-proteins L23 and L29 owing to their close proximity to the exit site where newly formed polypeptides emerge. Together these r-proteins form attachment sites for trigger factor (TF) [3, 4], a bacterial chaperone that binds to nascent peptide chains emerging from the ribosome, and signal recognition particle (SRP) [5, 6], a factor that coordinates the co-translational targeting of secretory and membrane proteins to the endoplasmic reticulum membrane in eukaryotes or the inner membrane in bacteria. Even though TF is only present in eubacteria and chloroplasts, the ability of L23 and L29 to form attachment sites for other important ribosome-associated factors appears to be universally conserved [7]. Interestingly, whereas L23 is essential for both TF binding and for the viability of *E. coli*, the neighboring L29 protein is not required for either [3]. In fact, all exit tunnel r-proteins (L4, L22 and L23) except for L29 are essential for *E. coli* viability, highlighting the critical role of these proteins in the synthesis and folding of nascent polypeptides [8]. At present, there is very little information regarding the role of r-protein L29 with respect to cellular protein biogenesis (e.g., protein synthesis, protein folding, secretory targeting, degradation, etc.). Therefore, the goal of this study was to address the extent to which L29 affects the expression of structurally diverse recombinant proteins.

## 2 Materials and methods

### 2.1 Bacterial strains and growth conditions

*E. coli* strains BL21(DE3) Δ*rpmC*::kan and BL21 (DE3) Δ*tig*::kan were constructed by P1 transduction of BL21(DE3) cells (Novagen) with the Δ*rpmC*::kan allele derived from donor strain MC4100 Δ*rpmC*::kan [3] and BB6515 (MC4100 Δ*tig*::kan) [9]. Strains were grown aerobically at 37°C in Luria-Bertani (LB) medium, and antibiotic supplements were at the following concentrations: ampicillin, 100 μg/mL; chloramphenicol, 25 μg/mL; kanamycin 50 μg/mL. Protein synthesis was induced with 1 mM IPTG and/or 0.2% arabinose when the cells reached an absorbance at 600 nm (*A*_600_) of ∼0.25–0.50. Samples to be analyzed were harvested following 4–6 h of induction.

### 2.2 Plasmid construction

All plasmids used in this study were constructed using standard cloning procedures. Briefly, plasmids for expressing the different recombinant proteins were created by PCR amplification of the gene of interest followed by ligation of the restriction digested PCR product between the *Nhe*I and *Hin*dIII sites of plasmid pET21a (Novagen). A plasmid for expressing L29 was constructed by PCR amplification of the *rpmC* gene encoding L29 from *E. coli* genomic DNA followed by ligation between the *Sac*I and *Xba*I sites of pBAD18. Error-prone PCR of the *rpmC* gene was performed according to established protocols to generate a 1.5% error-rate library [10]. Error-prone PCR products were ligated into pBAD18, electroporated into ElectroMAX DH10B™ cells (Invitrogen) and plated on ampicillin. Characterization of the library (e.g., number of members, diversity) was performed as described elsewhere [11]. Single amino acid substitutions were made in wild type (wt) L29 using the QuikChange® II site-directed mutagenesis kit (Stratagene) according to manufacturer's instructions.

### 2.3 Protein, DNA, and RNA analysis

To generate whole cell lysates and soluble fractions, 10-mL cultures were divided and pelleted by centrifugation for 15 min at 4°C and 3500 rpm. Whole cell lysates were prepared by washing the first pellet in PBS followed by centrifugation and resuspension of the pellet directly in 300 μL of SDS-PAGE loading buffer and heating for 10–15 min at 95°C. The soluble and insoluble fractions were prepared as described previously [12]. To ensure that an equivalent number of cells was analyzed, culture volumes were all normalized to the same *A*_600_. Plasmid DNA content in whole cell lysates was determined by isolating plasmid DNA from an equivalent number of wt or Δ*rpmC*::kan cells using a Qiagen miniprep kit and quantifying the concentration using a NanoDrop ND-1000 spectrophotomer. Activity of the plasmid-encoded resistance protein β-lactamase in soluble cell lysates from wt or Δ*rpmC*::kan cells was determined using a standard assay with nitrocefin as substrate. Absorbance at 486 nm was measured on a Molecular Device SpectraMax 190 spectrophotometer and initial rates were determined for the initial linear absorbance change [13, 14]. 70S Ribosomes were isolated according to standard methods as described elsewhere [12]. Total RNA was extracted from cells using an RNeasy Mini Kit (Qiagen) according to the manufacturer's instructions and transcript abundance was determined by qRT-PCR as described elsewhere [15].

For green fluorescent protein (GFP) activity, 3 μL whole cell lysate or soluble fraction was resuspended in 100 μL PBS, added to a 96-well plate. Samples were assayed using a fluorescence microplate reader (BioTek Synergy HT) and the resulting fluorescence was normalized by the total amount of protein detected in each sample via the Bradford assay. For GFP activity of intact cells, fluorescence microscopy or flow cytometry using a Becton Dickinson FACSCalibur in scan mode was performed as described elsewhere [11]. FACS-based screening of bacterial cell libraries was performed as described previously [11]. β-Galactosidase (β-gal) activity was determined using the standard Miller assay and scFv13-R4 activity was determined by standard ELISA with β-gal as antigen [12, 16]. Proteins were resolved by SDS-PAGE using 12% Tris-HCl gels and immunoblotted according to standard protocols. The following primary antibodies were used with the corresponding dilution in parenthesis: mouse anti-GFP (1:2000; Sigma); mouse anti-MBP (1:4000; Sigma), mouse anti-TrxA (1:1000; Stratagene); mouse anti-β-gal (1:500; Abcam); mouse anti-FLAG M2 (1:1000; Stratagene); and rabbit anti-TF. To verify the quality of subcellular fractions, membranes were probed with mouse anti-GroEL antibodies (1:10 000; Sigma). For immunoblotting of 70S ribosomal fractions, 70S fractions were normalized by the amount of rRNA as determined by measuring *A*_260_.

## 3 Results and discussion

**Figure 1:**
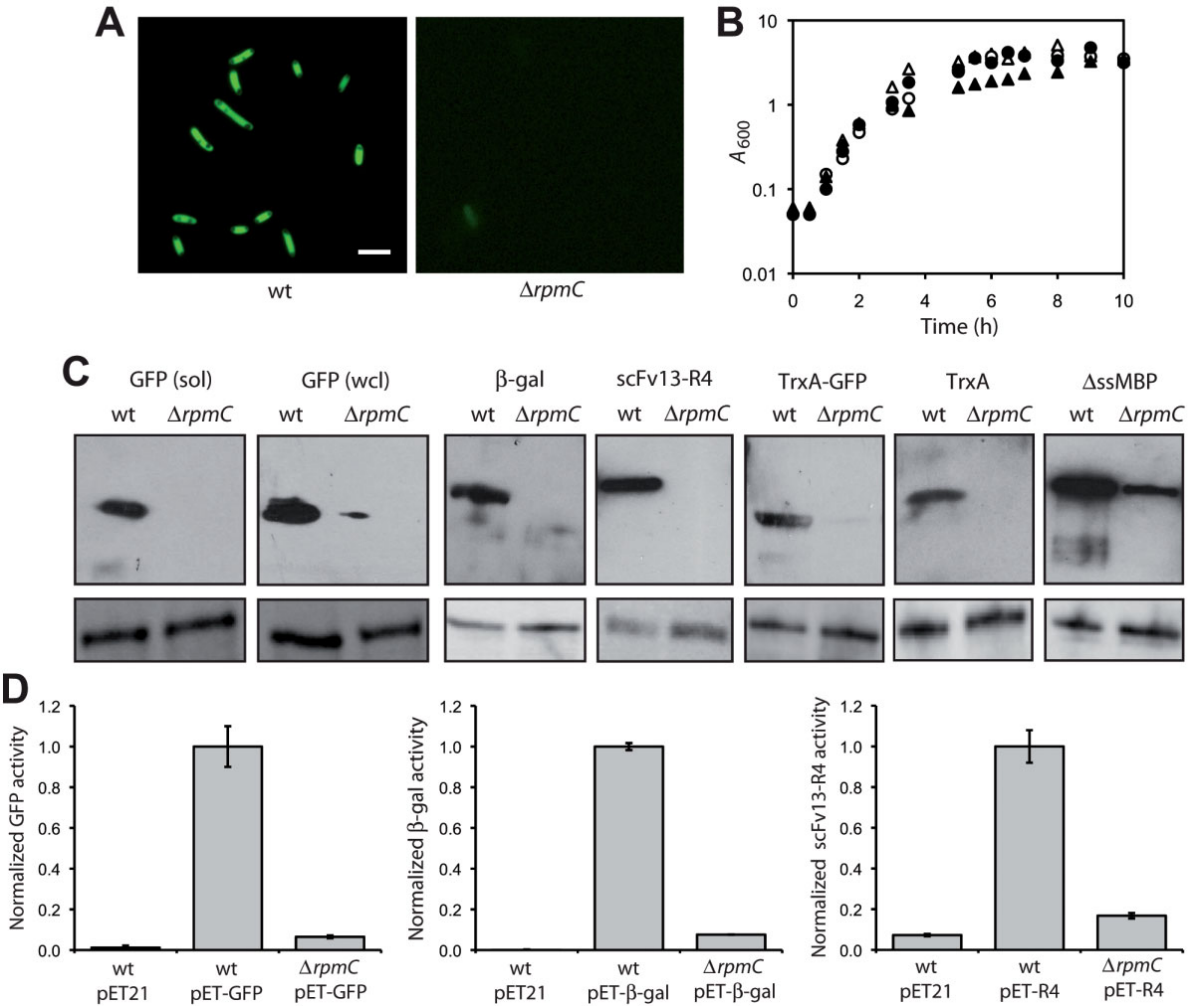
Expression of recombinant proteins in L29-deficient cells. (**A**) Fluorescence microscopy and (**B**) cell growth of wt and Δ*rpmC*::kan cells expressing GFP. Scale bar, 1 μM. Growth curves show induced (closed) and uninduced (open) cultures of wt (triangle) or Δ*rpmC*::kan (circle) cells. Data show the average of three independent experimental repeats and the SEM for these data was less than 5%. (**C**) Western blot analysis of whole cell lysates (wcl) isolated from wt and Δ*rpmC*::kan cells expressing different target proteins as indicated. For GFP, the soluble (sol) fraction is also shown. An equivalent number of cells was loaded in each lane. GroEL served as a loading control (lower panels). (**D**) Activity of GFP, β-gal and scFv13-R4 measured in whole cell lysates prepared from wt and Δ*rpmC*::kan cells carrying an empty vector control (pET) or a pET vector with the indicated protein. GFP activity was measured by FACS and cells were ungated, β-gal activity was measured using the Miller assay, and scFv13-R4 activity was determined by ELISA. All values were normalized to the activity measured in wt cells. An equivalent number of cells was assayed in each case. Data show the average of at least three independent experimental repeats and the error bars represent the SEM.

We hypothesized that r-proteins L29 plays a key role in cellular protein production. The rationale for this hypothesis is the close proximity of L29 to (i) well-defined docking sites for chaperones that are important for protein folding and (ii) other tunnel proteins that are essential for translation and cell viability. To test this hypothesis, we explored whether the lack of L29 affected the expression of different prokaryotic and eukaryotic proteins in vivo. Starting with GFP, we immediately noticed that *E. coli* BL21(DE3) cells lacking L29 accumulated little to no active GFP whereas wt BL21(DE3) cells expressing the same construct were highly fluorescent (Fig. 1A). Western blot analysis confirmed that GFP was completely absent from the soluble fraction prepared from cells that lacked L29, but accumulated to a high level in the soluble fraction prepared from wt cells (Fig. 1C). Whole cell lysates prepared from the Δ*rpmC*::kan strain were also largely devoid of GFP (Fig. 1C), suggesting that GFP was either not sufficiently synthesized or was rapidly degraded in these cells. We favored the former possibility because there were no detectable bands corresponding to degraded GFP in any of the Western blots. It should be noted that the absence of GFP was not explained by misfolding and/or aggregation as very little GFP was detected in the insoluble fraction of both strains (data not shown). Consistent with the microscopy and Western blot results, GFP activity was significantly lower in the soluble fraction and whole cell lysates derived from Δ*rpmC*::kan cells compared to the same fractions isolated from wt cells (Fig. 1D, shown for whole cell lysates). Despite this disparity in GFP expression, there was no significant difference in growth for wt and Δ*rpmC*::kan cells in the presence or absence of inducer (Fig. 1B). A small but reproducible growth decrease was observed for wt cells following induction of GFP expression but these cells eventually reached the same final density of the other cells. Thus, the weak expression of GFP observed in L29-deficient cells was apparently not the result of a general growth defect.

To determine if this phenomenon was specific to GFP, we analyzed the expression of two additional proteins, *E. coli* β-gal and a single-chain Fv antibody fragment (scFv13-R4) specific for β-gal that was previously optimized for cytoplasmic expression in *E. coli* [17]. Similar to the case with GFP, the β-gal and scFv13-R4 proteins were only detected in wt cells but not in cells lacking L29 (Fig. 1C). Also, as with GFP, there was no direct evidence for degradation or aggregation of either of these proteins in Δ*rpmC*::kan cells. In agreement with the Western blot analysis, the activity of the β-gal and scFv13-R4 proteins was significantly lower in the Δ*rpmC*::kan cells compared to wt cells (Fig. 1D). Thus, the absence of L29 negatively affects the expression of proteins other than GFP including β-gal, which is native to *E. coli*.

We next tested whether defective GFP synthesis in Δ*rpmC*::kan cells could be rescued by fusion to thioredoxin (TrxA) or maltose-binding protein (MBP), both of which are well known to improve the expression of proteins to which they are fused [18]. A chimera between TrxA and GFP was efficiently expressed in wt cells with no visible degradation (Fig. 1C). Moreover, expression of the TrxA-GFP chimera in wt cells resulted in higher GFP fluorescence relative to expression of unfused GFP (data not shown), consistent with the ability of TrxA to enhance the expression of its fusion partner. However, similar to the case with unfused GFP, TrxA-GFP was hardly detectable in cells lacking L29 (Fig. 1C). We observed a similar result when GFP was expressed in the cytoplasm as a fusion to MBP lacking its native export signal (ΔssMBP; data not shown). In fact, even the unfused TrxA and ΔssMBP proteins were expressed very poorly in Δ*rpmC*::kan cells (Fig. 1C), which was surprising given that these are native to *E. coli* and typically express very well [13, 19, 20]. Indeed, TrxA and especially MBP were efficiently expressed in wt cells (Fig. 1C). Taken together, these data show that the protein expression defect observed in cells lacking L29 affects structurally diverse proteins of different origin, and cannot be overcome by modifications to the target protein that enhance its expression and/or folding efficiency.

We next sought to determine the cause of the protein expression deficiency in cells lacking L29. First, we investigated whether the low intracellular accumulation of GFP in Δ*rpmC*::kan cells was caused by the absence of an accessory that normally binds to ribosomes. In particular, a loop region located between helices α1 and α2 of L29 is known to form part of the scaffold for the docking of the molecular chaperone TF to ribosomes [21]. As a result, TF associates in very close proximity to the ribosomal exit tunnel and may participate in GFP expression by creating a protected folding space for the emerging nascent chain. To test this hypothesis, we investigated whether TF was still associated with ribosomes lacking L29. As shown in Supporting information, Fig. S1A, the amount of TF associated with 70S ribosomes in wt and Δ*rpmC*::kan cells was nearly identical. This result was consistent with the observation that L29 is not essential for TF binding to ribosomes [3] and suggests that the mechanism by which GFP expression is decreased in L29-deficient cells does not depend on the recruitment and association of TF to 70S ribosomes. One possibility that we cannot rule out is that TF bound to L29-deficient ribosomes interacts less efficiently with GFP (and other nascent polypeptides) compared to TF bound to wt ribosomes. This notion is supported by the finding that L29 induces a conformational change in TF that exposes hydrophobic patches of TF to the opening of the ribosomal exit tunnel, thereby increasing its affinity for hydrophobic segments of emerging nascent polypeptides [4]. However, it seems unlikely that a change in TF activity is responsible for the poor GFP expression because cells that lacked L29 did not accumulate insoluble GFP or exhibit increased GFP degradation, both of which are hallmarks of protein misfolding in *E. coli*.

**Figure 2:**
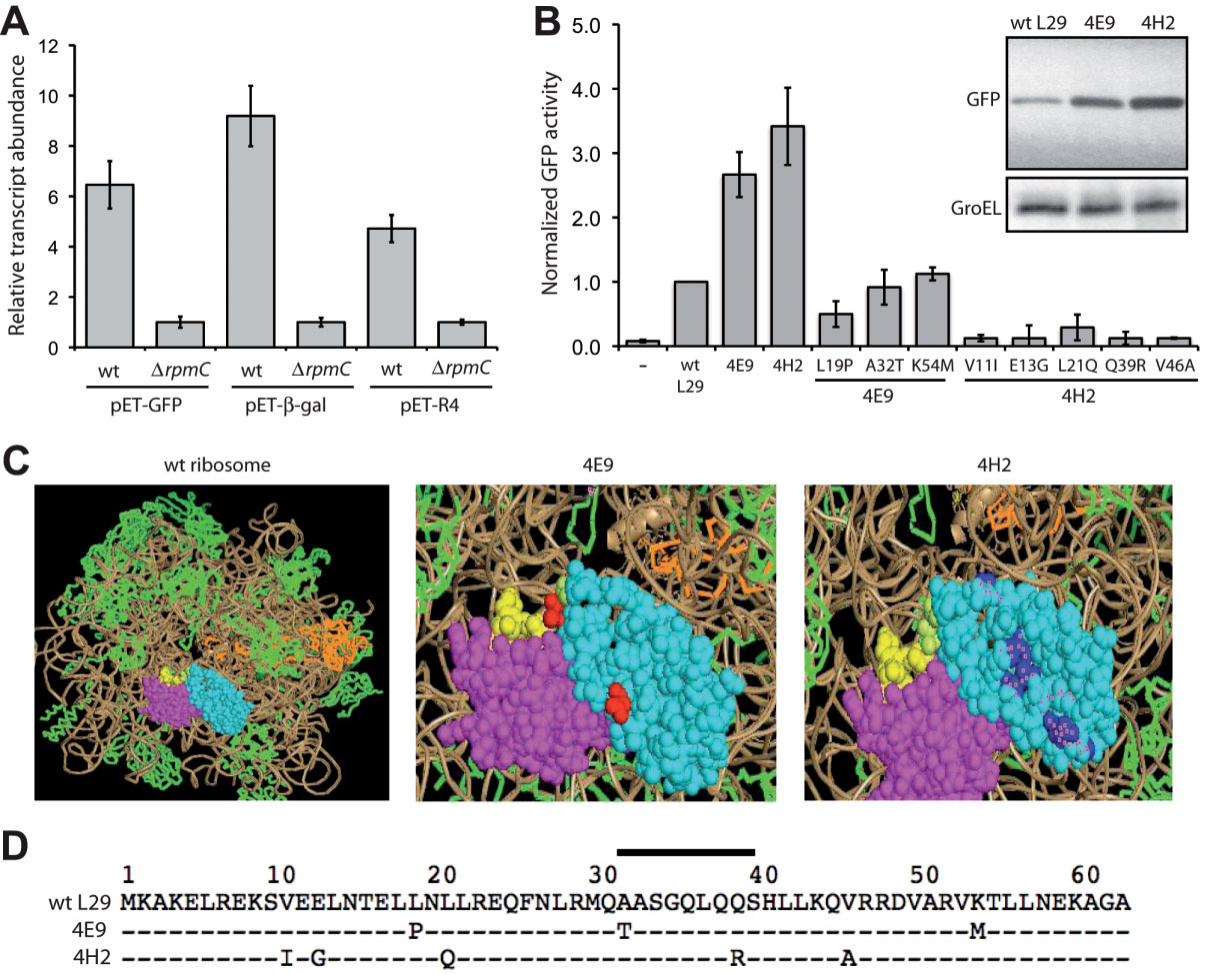
Characterization and engineering of GFP expression in L29-deficient cells. (**A**) qRT-PCR analysis of mRNA transcript levels for GFP, β-gal and scFv13-R4. RNA was isolated from wt and Δ*rpmC*::kan cells carrying the plasmid indicated. All data was normalized to the amount of 16S rRNA measured in each strain. Relative induction was calculated by dividing each normalized value by the value measured in Δ*rpmC*::kan cells expressing GFP, β-gal, or scFv13-R4. Data show the average of six independent experimental repeats and the error bars represent the SEM. (**B**) Cellular GFP activity produced by Δ*rpmC*::kan cells in the presence of empty plasmid (–), wt L29, or the L29 variants 4E9, 4H2, and the single amino acid substitution mutants as indicated. Cell fluorescence was measured by FACS and cells were ungated. Values were normalized to control cells co-expressing GFP and wt L29. Data show the average of three independent experimental repeats and the error bars represent the SEM. Inset shows western blot analysis of whole cell lysates isolated from Δ*rpmC*::kan cells co-expressing GFP and different L29 variants as indicated. GroEL served as a loading control. (**C**) Structural depiction of wt and mutant L29 proteins (cyan) in the context of L23 (magenta) and the TF-binding site (yellow). Mutations in 4E9 and 4H2 are shown in red and blue, respectively. (**D**) Amino acid sequence alignment of *E. coli* L29 and related variants. Bar indicates the TF-binding region.

Thus, we next explored whether the absence of GFP in Δ*rpmC*::kan cells was due to a defect in an earlier stage of protein biosynthesis. Specifically, we measured the levels of *gfp* mRNA in wt and Δ*rpmC*::kan cells. To our surprise, a ∼6.5-fold decrease in the *gfp* mRNA transcript levels was observed for cells lacking L29 relative to wt cells (Fig. 2A). Hence, the dramatic decrease in GFP protein levels seen for Δ*rpmC*::kan cells relative to wt cells appears to be due in part to an unexpectedly large decrease in *gfp* mRNA abundance. Similarly large decreases in transcript abundance were also observed for β-gal and scFv13-R4 (Fig. 2A), suggesting that the same mechanism was affecting the expression of these proteins as well. To rule out that RNA polymerase, or Pol I, which is specific for plasmid replication, was not suffering from lower expression in this system, we measured the cellular plasmid content and the cellular activity of the resistance gene product β-lactamase (Supporting information, Fig. S1B). From these experiments, it is clear that the amount of plasmid is the same in wt and L29 mutants.

At present, the details describing how cells lacking L29 down-regulate the expression of these plasmid-encoded genes remain unclear; however, this must occur by either lowered mRNA transcription or increased mRNA degradation. It has been reported that, although ribosomes do not affect the total levels of mRNA that are transcribed, their binding to mRNA transcripts in bacteria provides stability, perhaps by steric protection from cellular RNases [22]. Thus, an intriguing hypothesis is that L29-deficient ribosomes may be less efficient at binding and protecting mRNA transcripts from degradation, thereby resulting in a decrease in the mRNA transcript abundance. An alternative possibility is that cells lacking L29 exhibit a moderate defect in translation that is nearly undetectable under normal conditions, but becomes much more pronounced during high-level expression of plasmid-encoded genes as L29-deficient ribosomes may not be able to keep pace with the biosynthetic demands placed on the cell. To ensure translational quality control in L29-deficient cells where synthesis of plasmid-encoded genes may be compromised, A-site mRNA cleavage and the tmRNA system may provide a mechanism for reducing translational errors and the production of aberrant and potentially harmful polypeptides [23]. A final possibility is that lack of L29 creates an altered ribosomal exit tunnel that somehow occludes the nascent polypeptide, thereby resulting in elongation arrest, which in turn induces either A-site cleavage or other modes of cleavage of the transcript [24]. While we do not see alterations in the levels of a housekeeping factor, 16S rRNA, in wt and Δ*rpmC* cells, we cannot rule out the possibility that altered mRNA levels for other cellular proteins may be partly responsible for the changes in target protein expression seen here and is a topic that warrants further investigation.

Finally, to explore the limits of the ability of L29 to promote high-level protein expression, we used rounds of mutagenesis and in vivo screening to identify L29 variants that increased expression of GFP. Specifically, Δ*rpmC* cells co-expressing GFP with an error-prone library of L29 genes were screened by FACS to isolate highly fluorescent bacteria. After screening ∼1 × 10^7^ bacterial clones, the variants 4E9 and 4H2 were isolated. Co-expression of these variants with GFP in Δ*rpmC* cells resulted in a 2.7- and 3.4-fold increase, respectively, in cellular GFP activity relative to Δ*rpmC* cells expressing wt L29 (Fig. 2B). Interestingly, whereas no growth difference was observed between wt and Δ*rpmC* cells (Fig. 1B), expression of clone 4E9 promoted a reproducible ∼three-fold increase in growth rate (data not shown). In agreement with the increase in GFP activity, Western blot analysis indicated that GFP accumulation in cells co-expressing 4E9 or 4H2 was clearly increased (Fig. 2B, shown for 4H2). Inspection of the location of the 4E9 and 4H2 mutations in the context of *E. coli* ribosomes indicates that most of the mutations occurred on the surface of ribosomes near the exit tunnel (Fig. 2C and D). Thus, it is tempting to speculate that these mutations improve GFP expression by productively interacting with the emerging nascent GFP polypeptide or by positively altering the activity of an exit tunnel accessory factor. Interestingly, all of the mutations in our best clone 4H2 are observed on an individual basis in different bacterial genomes (Supporting information, Fig. S2). Moreover, two of the 4H2 mutations (L21Q, V46A) occur together in the genome of Acinetobacter. As expected, no single L29 protein in nature carries all five of the 4H2 mutations simultaneously. A decoupling analysis for mutants 4E9 and 4H2 was also performed, where each of the amino acid substitutions was analyzed independently. From this analysis, we conclude that the mutations found in 4E9 and 4H2 must act synergistically to confer the observed phenotype because none of the single point mutations in L29 were able to increase GFP expression (Fig. 2B). It should also be noted that the 4H2 mutant was able to increase the expression of β-gal and scFv13-R4 proteins (Supporting information, Fig. S1C), indicating that this mutant was not specific to GFP and perhaps a more general expression-enhancing factor. Based on these results, we anticipate that the application of directed evolution to the translational machinery, especially exit tunnel proteins like L23 and L29, holds untapped potential for creating tailor-made ribosomes that are capable of efficiently synthesizing complex target proteins. This is reminiscent of recent studies where directed evolution was used to engineer molecular chaperone systems in *E. coli* that aided the folding of eukaryotic proteins such as GFP and firefly luciferase [25, 26].

## 4 Concluding remarks

Here, we demonstrated that the synthesis of several diverse proteins including GFP becomes down-regulated in *E. coli* cells whose ribosomes lacked L29. We also isolated L29 variants that promoted increased accumulation of GFP. Taken together, these data support the notion that the ribosomal exit tunnel is not simply a static conduit for newly made proteins but rather a dynamic channel that is intimately involved in the production of cellular proteins. We anticipate that improved knowledge of how exit tunnel proteins contribute to protein expression and folding could be key in the context of rationally engineering bacterial translation for efficient production of active, structurally complex proteins.

## Abbreviations

bgr;-gal: β-galactosidase
FACS: fluorescence activated cell sorting
GFP: green fluorescent protein
MBP: maltose-binding protein
mRNA: messenger ribonucleic acid
r-Proteins: ribosomal proteins
rRNA: ribosomal ribonucleic acid
scFv: single-chain Fv antibody
TrxA: thioredoxin
TF: trigger factor
wt: wild-type

## Supporting information

Detailed facts of importance to specialist readers are published as ”Supporting Information”. Such documents are peer-reviewed, but not copy-edited or typeset. They are made available as submitted by the authors.
