# Supplementary material for: The ribosomal exit tunnel as a target for optimizing protein expression in Escherichia coli
Source: Biotechnol J. 2011 Nov 11;7(3):354–60. doi: 10.1002/biot.201100198 (PMC3382190; doi:10.1002/biot.201100198)
Supplement: Supplementary file 1 [file biot0007-0354-SD1.pdf]

Biotechnology
Journal

Supporting Information for DOI 10.1002/biot.201100198

The ribosomal exit tunnel as a target for optimizing protein expression in *Escherichia coli*

Lydia M. Contreras-Martin, Jason T. Boock, Jan S. Kostecki and Matthew P. DeLisa

© 2011 Wiley-VCH Verlag GmbH & Co. KGaA, Weinheim

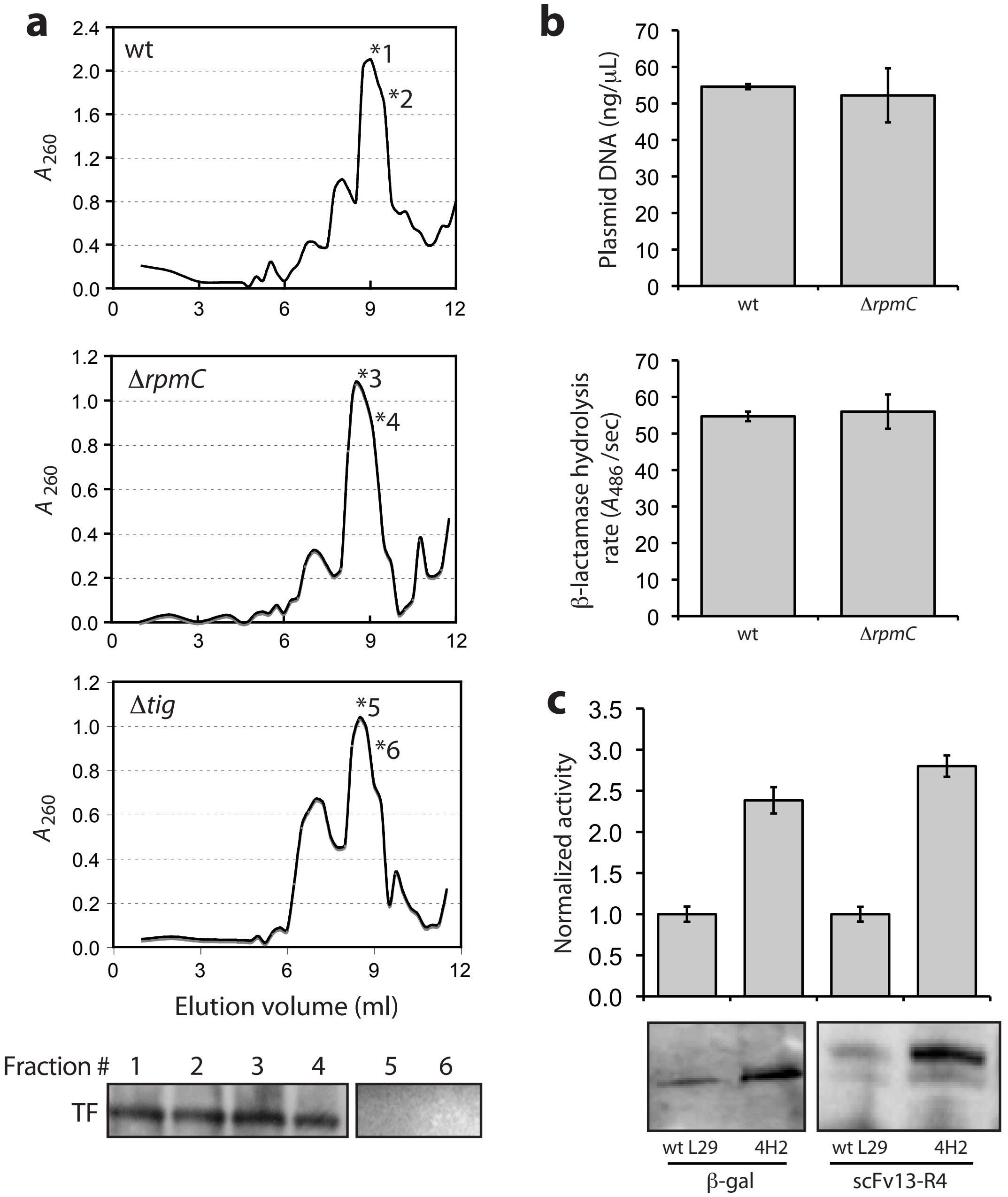

Supplemental Figure S1. Additional characterization and engineering of GFP expression in L29-deficient cells. (a) RNA concentration in sucrose gradient fractions from wt cells (top) and Δ*rpmC*::kan cells (middle) as determined by OD_{260} measurements. Fractions from Δ*tig*::kan cells (bottom) lacking TF were included as a negative control. Asterisks denote the fractions containing 70S ribosomes that were used for immunoblotting analysis, which is shown in the lower panel. Western blots were probed with anti-TF antibodies. An equivalent number of ribosomes was loaded in each lane. **(b)** Quantification of DNA concentration and β-lactamase activity in whole cell lysates prepared from wt and Δ*rpmC*::kan cells. For DNA quantification, plasmid DNA was isolated from whole cells using a Qiagen miniprep kit according to manufacturer's instructions and quantified using a NanoDrop ND-1000 spectrophotometer. For β-lactamase activity, samples were analyzed using a standard spectrophotometric assay with nitrocefin as substrate. Whole cell lystates were prepared from an equivalent number of cells. **(c)** Activity of GFP, β-gal and scFv13-R4 measured in soluble fractions prepared from wt and Δ*rpmC*::kan cells carrying an empty vector control (pET) or a pET vector with the indicated protein. GFP activity was measured by FACS and cells were ungated, β-gal activity was measured using the Miller assay, and scFv13-R4 activity was determined by ELISA. All values were normalized to the activity measured in wt cells. An equivalent number of cells was assayed in each case. Data is the average of at least three independent experimental repeats and the error bars represent the SEM. Western blot analysis of whole cell lysates (wcl) isolated from wt and Δ*rpmC*::kan cells expressing different target proteins as indicated. For GFP, the soluble (sol) fraction is also shown. An equivalent number of cells was loaded in each lane. GroEL served as a loading control (lower panels).

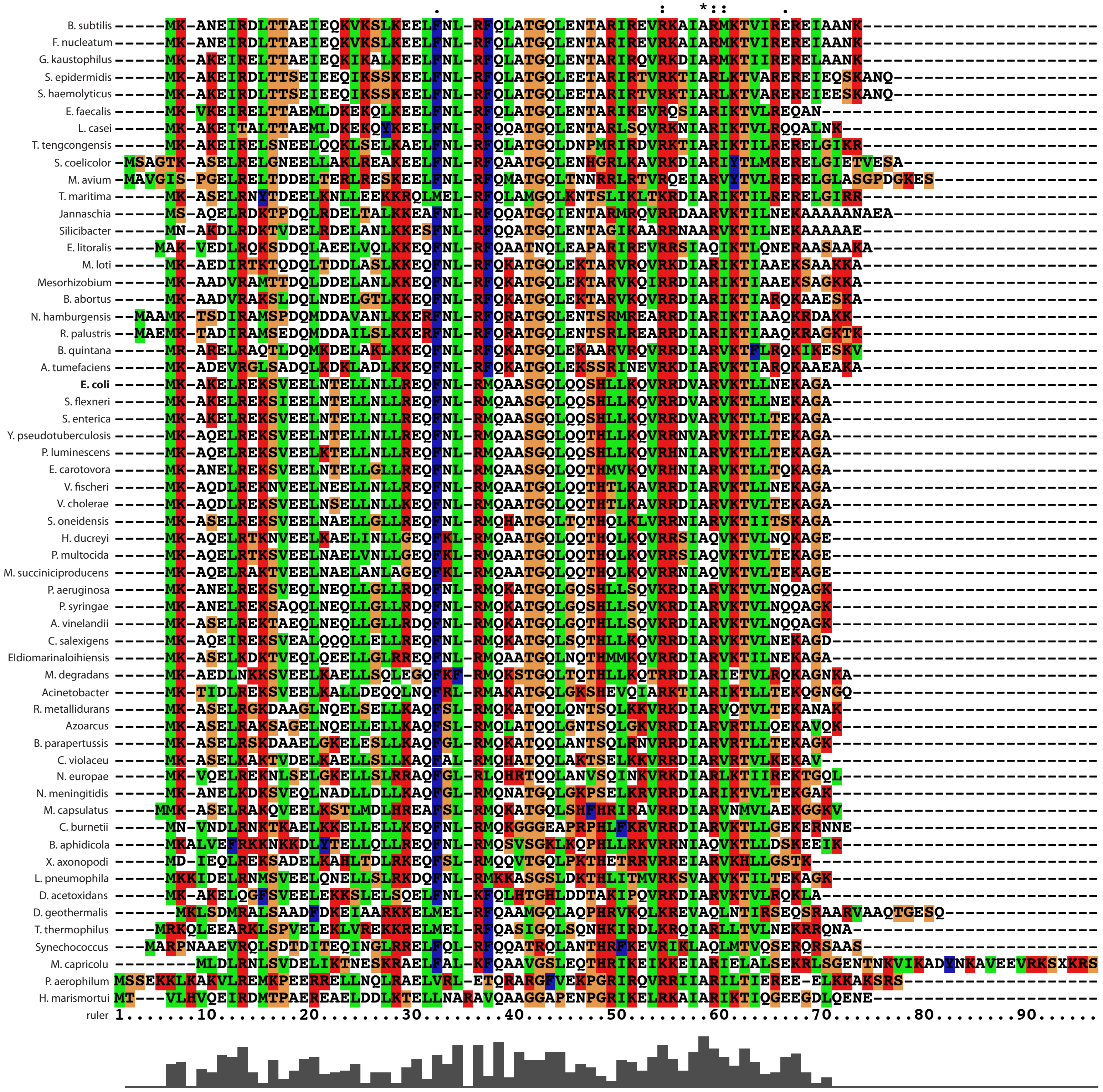

Supplemental Figure S2. Multiple sequence alignment of bacterial L29 proteins. Figure generated using CLUSTALX (1.81.1-alpha) multiple sequence alignment tool. The mutations in mutant 4H2 that are also found in nature include: V11I (naturally observed in *Shigella*); E13G (naturally in *Ralstonia metallidurans*); L21Q (naturally in *Acinetobacter* and *Microbulbifer degradans*); Q39R (naturally in *Coxitella burnetii*); V46A (naturally in *Silicibacter*, *Nitrobacter hamburgensis*, *Rhodopseudomonas palustris*, *Acinetobacter*). Note that two 4H2 mutations occur naturally in *Acinetobacter*.
